# Use of Electrospun Phenylalanine/Poly-ε-Caprolactone Chiral Hybrid Scaffolds to Promote Endothelial Remodeling

**DOI:** 10.3389/fbioe.2021.773635

**Published:** 2021-11-25

**Authors:** Benlin Sun, Lei Hou, Binbin Sun, Yu Han, Yunqing Zou, Juexin Huang, Yanan Zhang, Chuanliang Feng, Xiaoqiu Dou, Feng Xu

**Affiliations:** ^1^ Guangxi Collaborative Innovation Center for Biomedicine, Guangxi Medical University, Nanning, China; ^2^ Department of Cardiology, Tongren Hospital, School of Medicine, Shanghai Jiao Tong University, Shanghai, China; ^3^ Department of Orthopaedic Surgery, Shanghai Key Laboratory of Orthopaedic Implants, Shanghai Ninth People’s Hospital, Shanghai Jiao Tong University School of Medicine, Shanghai, China; ^4^ Clinical and Translational Research Center for 3D Printing Technology, Medical 3D Printing Innovation Research Center, Shanghai Ninth People’s Hospital, Shanghai Jiao Tong University School of Medicine, Shanghai, China; ^5^ State Key Lab of Metal Matrix Composites, School of Materials Science and Engineering, Shanghai Jiao Tong University, Shanghai, China; ^6^ Department of Subject Planning Shanghai, Ninth People’s Hospital Shanghai, Jiao Tong University School of Medicine, Shanghai, China

**Keywords:** tissue-engineered vascular graft, scaffold, electrospinning, chirality, endothelial remodeling

## Abstract

The fabrication of tissue-engineered vascular grafts to replace damaged vessels is a promising therapy for cardiovascular diseases. Endothelial remodeling in the lumen of TEVGs is critical for successful revascularization. However, the construction of well-functioning TEVGs remains a fundamental challenge. Herein, chiral hybrid scaffolds were prepared by electrospinning using D/L-phenylalanine based gelators [D(L)PHEG] and poly-ε-caprolactone (PCL). The chirality of scaffolds significantly affected the endothelial remodeling progress of TEVGs. Compared with L-phenylalanine based gelators/poly-ε-caprolactone (L/PCL) and PCL, D-phenylalanine based gelators/poly-ε-caprolactone (D/PCL) scaffolds enhanced cell adhesion, and proliferation and upregulated the expression of fibronectin-1, and vinculin. These results suggests that chiral hybrid scaffolds can promote endothelial remodeling of TEVGs by upregulating adhesion-associated protein levels. This study offers an innovative strategy for endothelial remodeling of TEVGs by fabricating chiral hybrid scaffolds, and provides new insight for the treatment of cardiovascular diseases.

## Introduction

Cardiovascular diseases are associated with extremely high morbidity and mortality rates in various countries worldwide, and the primary threat to human health globally (Bao et al., 2021). Owing to the risk of cardiovascular diseases, their treatment methods have always been an area of concern. Using autologous blood vessels or artificial blood vessel grafts to replace damaged blood vessels is a promising method used for treatment. However, the number of blood vessels that can be used as autologous transplants is limited, specifically in patients with cardiovascular diseases (Rayatpisheh et al., 2014; Wang et al., 2020). Therefore, the use of artificially synthesized blood vessels to replace damaged blood vessels that cannot be regenerated is of great significance in cardiovascular diseases (Badhe et al., 2017). Consequently, TEVGs have received widespread attention (Radke et al., 2018; Wang and Weiss, 2019; Xie et al., 2019). However, current strategies for the transplantation of TEVGs still suffer from failure due to vascular stenosis, thrombosis, and other symptoms (Stowell and Wang, 2018; Kuang et al., 2020). To decrease the rate of the noted symptoms in transplants, endothelial remodeling of TEVGs must be promoted (Chang et al., 2016; Yi et al., 2020). Furthermore the progress of endothelial remodeling is regulated by many factors.

The speed of endothelial remodeling in the lumen primarily depends on the physiochemical properties and bioactivity of the materials used to fabricate TEVGs. Synthetic polymers such as poly-ε-caprolactone (PCL) have been extensively employed in electrospinning for the fabrication of tissue-engineering vessels because of their excellent biocompatibility and mechanical properties (Zhao et al., 2018; Iglesias-Echevarria et al., 2019; Yang et al., 2019). Electrospinning is a unique technique used for fabrication of tissue-engineered vessels with nanofibrous topography and has great potential for mimicking the extracellular matrix. The properties of scaffolds fabricated by electrospinning can be tuned by changing the parameters and mixture (Hasan et al., 2014; Wang and Weiss, 2019). However, synthetic polymers still require functional modification because of absence of bioactive sites for cell binding and intrinsic hydrophobicity (Zhao et al., 2018). Physical methods of surface modification, such as plasma deposition, plasma polymerization, radiation grating, surface fixation, etc., are used to improve cell interactions. Surface modification is only a process of passive cell adhesion of the signals required for cell survival. Therefore, synthetic polymers are often integrated with other materials to improve their properties (Annabi et al., 2011; Fu et al., 2014; Lee et al., 2016). Despite excellent short-term results, the reinforced vascular graft fabricated using composite materials the reinforced vascular graft fabricated using composite materials lacks long-term survival or stability (Emechebe et al., 2020). Combining synthetic materials with other materials is still an essential problem to solve. Therefore, there is an urgent requirement to develop new strategies for endothelial remodeling of tissue-engineered vessels.

The endothelial remodeling process is directed by the extracellular microenvironment, including proteins, cytokines, structures, and mechanical and chiral properties (Hauser et al., 2017). Among these, the chiral structure plays a crucial role in vascular endothelium remodeling and has not been studied in depth. Chirality is a ubiquitous structure that is a distinctive signature that can be attributed to amino acids, proteins, and nucleotides in living organisms (Liu et al., 2015; Xing and Zhao, 2018). Chiral hydrogels have been proven to play an important role in cell differentiation (Arslan et al., 2017). The chiral molecule C2-symmetric D/L-phenylalanine-based gelators [D(L)PHEG] (Supplementary Figure S1) is a type of chiral hydrogel, and has a novel chiral nanofibrous structure. Previous research has found that the chiral hydrogels DPHEG and LPHEG influence cell behavior (Liu et al., 2018; Dou et al., 2020). It was also proved that those materials allow the differentiation of stem cells to different lineages of osteogenic and adipogenic cells (Wei et al., 2019). DPHEG nanofibers with right-handed helical structures enhance retinal progenitor cell differentiation compared with left-handed LPHEG nanofibers (Sun et al., 2021). In addition, chirality can influence the functions of many molecules. Switching the chirality of peptide moieties affects the degradation rate of enzymatic degradation by endogenously present enzymes (Griffin et al., 2020). Accordingly, chirality has been considered an important factor in the development of biomaterials for regular cell behavior. Therefore, by changing the chirality of the extracellular microenvironment, endothelial remodeling can be promoted to form TEVGs. Although the regulation of chiral structures for cells has been studied extensively, the chiral molecules used for electrospun scaffolds still need to be explored.

**FIGURE 1:**
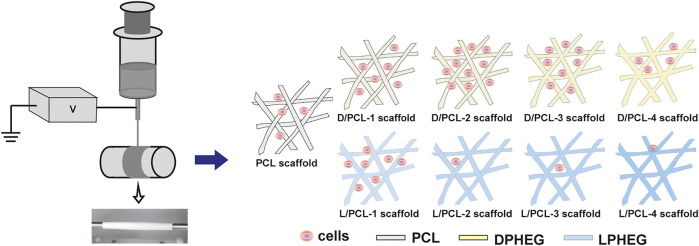
Schematic of the electrospinning process. Chiral hybrid scaffolds (D/PCL-1, D/PCL-2, D/PCL-3, D/PCL-4, L/PCL-1, L/PCL-2, L/PCL-3, and L/PCL-4 scaffolds) at the concentration of D(L)PHEG (1, 2, 3, and 4 mg/ml) were fabricated by blend electrospinning using PCL and D(L)PHEG. Different chiral hybrid scaffolds induced varying influences on endothelium remodeling.

In this study, chiral hybrid scaffolds were fabricated by electrospinning using solvent-dependent chiral assemblies of C2-symmetric D(L)PHEG and PCL, that mimic the extracellular microenvironment of human vascular endothelial cells (HUVECs) (Figure 1). First, we explored the effects of chirality on the extracellular microenvironment. DPHEG hydrogels enhanced considerably the adhesion and proliferation of HUVECs. Subsequently, chiral hybrid scaffolds were used to explore further the influence of chirality on endothelial remodeling. Biological assays showed that HUVECs on D/PCL scaffolds can form firm cell–scaffold/cell–cell interactions, while fibronectin-1, and vinculin were upregulated. This study revealed the influences of chiral molecules on endothelial remodeling of tissue-engineered vessels. These studies are expected to provide a new strategy for the preparation of tissue-engineered vessels.

## Materials and Methods

### Materials Synthesis

All starting materials and solvents were obtained from commercial suppliers and used without further purification. L/D-phenylalanine methyl ester hydrochloride, 1,4-benzenedicarbonyl dichloride and diglycol were purchased from Aladdin Chemicals. LPHEG and its enantiomer DPHEG were synthesized according to a previous report (Dou et al., 2019). ^1^H NMR (400 M Hz, DMSO-d_6_, δ) results are as follows: δ = 3.1 (m, 4H, CH_2_), 3.4 (m, 16H, CH_2_), 4.2 (q, 2H, OH), 4.7 (q, 2H, CH), 7.3 (m, 10H, Ar H), 7.8 (s, 4H, Ar H), 8.9 (d, 2H, NH) ppm. EI-MS for L(D)PFEG calcd. 636.71; found 637.28 (M + H)^+^.

### Hydrogel Preparation

The D(L)PHEG powder was suspended in deionized water (2 mg/ml). The solution was then heated until a transparent solution was obtained. The hydrogel was formed when the solution was cooled down to 25°C.

### Fabrication of Chiral Hybrid Scaffolds

PCL (MW 80 000, Sigma–Aldrich, United States ) and D(L)PHEG were dissolved in 1,1,1,3,3,3-hexafluoro-2-propanol (HFIP) to prepare the D(L)PHEG and PCL mixture. PCL was dissolved at a concentration of 12% w/v at different LPHEG or DPHEG concentrations at 1, 2, 3, and 4 mg/ml. Moreover, 12% w/v PCL/HFIP was prepared as a control. An electrospinning platform was used to generate chiral hybrid scaffolds, which consisted of a grounded collector covered by an aluminum foil, a high-voltage power supply and a syringe driven by a syringe pump. Electrospinning was conducted at an applied voltage of 10.0 kV, solution flow rate of 1.0 ml/h, needle tip-to-collector gap distance of 20 cm, and a collection speed of 200 revolutions per minute. Each of the scaffolds was collected on the aluminum foil after 6 h of electrospinning followed by drying in vacuum.

### Circular Dichroism (CD) Spectroscopy

First, the DPHEG and LPHEG powders were suspended in deionized water (2 mg/ml) to form hydrogels. The DPHEG and LPHEG powders were also dissolved in HFIP (2 mg/ml). CD and UV-Vis signals of DPHEG hydrogel, LPHEG hydrogel, DPHEG HFIP solution, and LPHEG HFIP solution were collected using the JACSO J-815 CD spectrometer. The CD spectra of all samples were recorded in the UV region (200–400 nm) with a bandwidth of 0.5 nm.

### Fourier Transform Infrared (FTIR) Spectra and Attenuated Total Internal Reflectance Fourier Transform Infrared (ATR-FTIR)

FTIR spectra of the DPHEG and LPHEG powders and ATR-FTIR spectra of all chiral hybrid scaffolds were obtained using a Bruck EQUINOX55 instrument. The PCL scaffold was used as a control. The KBr disk technique was used for solid-state measurements to collect FTIR spectra from D(L)PHEG powder samples. Samples were scanned in the wavelength range between 4,000 and 600 cm^−1^ at intervals of 0.4821 cm^−1^. To obtain ATR-FTIR spectra, chiral hybrid scaffolds were scanned in the wavelength range of 4,000–600 cm^−1^ at an interval of 1.9285 cm^−1^.

### Water Contact Angle Measurements

The hydrophilicity of different chiral hybrid scaffolds and PCL was assessed based on water contact angle measurements using a JC 2000D optical contact angle meter (Zhongchen, Shanghai). Results are indicative of the surface wettability of the scaffold. The PCL scaffold was used as a control. The water droplet (volume: 2.0 μl) was placed on the surfaces of different scaffolds for 1 and 10 s, and the shapes of the water droplet were recorded by a camera. The static water contact angles of the different chiral hybrid scaffolds were also recorded. For each scaffold, the measurements were conducted three times at room temperature (25°C) and the results were averaged (*n* = 3).

### Scanning Electron Microscopy (SEM)

SEM measurements were performed using an FEI QUANTA 250 microscope. LPHEG and DPHEG samples were prepared by depositing dilute solutions of hydrogel on silicon wafers and by drying them in an oven at 40°C overnight. The chiral hybrid scaffolds and PCL scaffolds were measured directly. The operating voltage was 10 kV.

### Tensile Test

The mechanical properties of the chiral hybrid scaffolds and PCL scaffolds were characterized by tensile tests performed on a universal tester (H5 K-S, Hounsfield, United Kingdom). The samples used for tensile testing were cut into 1 cm wide and 5 cm long stripes, and the thickness of each sample was measured using an electronic digital micrometer. The samples were stretched at a constant tensile rate of 90 mm/min. The stress–strain curves of all scaffolds were plotted. Young’s modulus, fracture strain, and ultimate tensile stress were analyzed statistically (*n* = 5).

### Cell Cultures

Primary HUVECs were obtained from Shanghai Jiao Tong University in Shanghai. They were cultured in DEME/F12 (HyClone, United States ) and supplemented with 5% (v/v) fetal bovine serum and 1% (v/v) antibiotics at 37°C in an incubator with 5% CO_2_ in a humidified atmosphere. HUVECs were trypsinized for cell seeding. The 24-well plates were coated with DPHEG and LPHEG (100 μl). The plates and scaffolds were pre-sterilized by exposure to ultraviolet (UV) light, with the plates exposed for 40 min.

### Cell Viability

Cell proliferation was evaluated based on the cell counting kit-8 assay (CCK-8, Beyotime, Shanghai, China). HUVEC suspensions (1 × 10^5^ cells per well) were seeded on 96-well plates, which contained chiral hybrid scaffolds and PCL scaffolds. CCK-8 reagent was added according to the recommended measurements after 1–5 days of incubation. After incubation for 2 h, a microplate reader (Thermo Fisher, United States ) was used to measure the optical density at 450 nm (630 nm was used as a reference). The results were analyzed statistically (*n* = 3).

### Live–Dead Assay

HUVECs (1 × 10^5^ cells per well) were seeded on 24-well plates coated with DPHEG hydrogel and LPHEG hydrogel cultured in an incubator for 2 days. In addition, 24-well plates containing chiral hybrid scaffolds and PCL scaffolds at the same cell number were cultured for 5 days. Subsequently, according to the recommended measurements, each well was stained using a Calcein/PI Live/Dead Viability/Cytotoxicity Assay Kit (Beyotime, Shanghai, China) for 30 min. A fluorescence microscope (LEICA, Germany) was used to acquire images of the DPHEG hydrogel and LPHEG hydrogel for 2 days. Chiral hybrid scaffolds were observed using a confocal laser scanning microscope (TCS SP5 II, Leica, Germany) on days 1 and 5.

### Fluorescent Staining

HUVECs (5 × 10^4^ cells per well) were seeded in 48-well plates with chiral hybrid and PCL scaffolds cultured for 1 day. The cytoskeletal organization and cell nuclei of HUVECs on the different scaffolds after 1 day of culture were analyzed by fluorescent staining. Samples for fluorescent staining were washed three times with phosphate buffer solution (PBS). HUVECs were then fixed with 4% (v/v) paraformaldehyde solution for 30 min. Subsequently, according to the recommended measurements, each well was stained with FITC–phalloidin (Servicebio, Wuhan, China) (that stains the actin cytoskeleton) for 120 min at room temperature, and then incubated with 4,6-diamidino-2-phenylindole (DAPI) (Servicebio, Wuhan, China) (that stains live nuclei) at 25°C for 10 min. The stained samples were washed and visualized using a confocal laser-scanning microscope. Image J was used to calculate the number of cells.

### Real-Time Quantitative Reverse Transcription-Polymerase Chain Reaction (RT-qPCR)

The RNeasy Mini Kit (Qiagen GmbH, Germany) was used to extract ribonucleic acid from HUVECs cultured on the D/PCL-2 and PCL scaffolds according to the manufacturer’s specifications. A blank control (tissue culture plastic) was used. Reverse transcription was achieved using the PrimeScript™ RT reagent synthesis kit with gDNA Eraser (Perfect Real Time) (Takara, Japan) by MiniOptiCon real time PCR detection system (BioRad, United States). All operations were performed according to the manufacturer’s instructions. The 2× SYBR Green qPCR Master Mix (Low Rox) (Quanta Biosciences, United States) and ABI 7300 real-time PCR system (Thermo Fisher, United States) were used for RT-qPCR. The primer sequences used are listed in Supplementary Table S1. Glyceraldehyde-3-phosphate dehydrogenase served as an internal control. All fold changes in gene expression were normalized to those in the control group.

### Statistical Analysis

All quantitative data are expressed as mean ± standard deviation. Statistical analysis was conducted using one-way analysis of variance (ANOVA) and Student’s t-test with *p* < 0.05 accepted as statistically significant.

## Results

### Characterization and Cell Viability of Chiral Hydrogel

**FIGURE 2:**
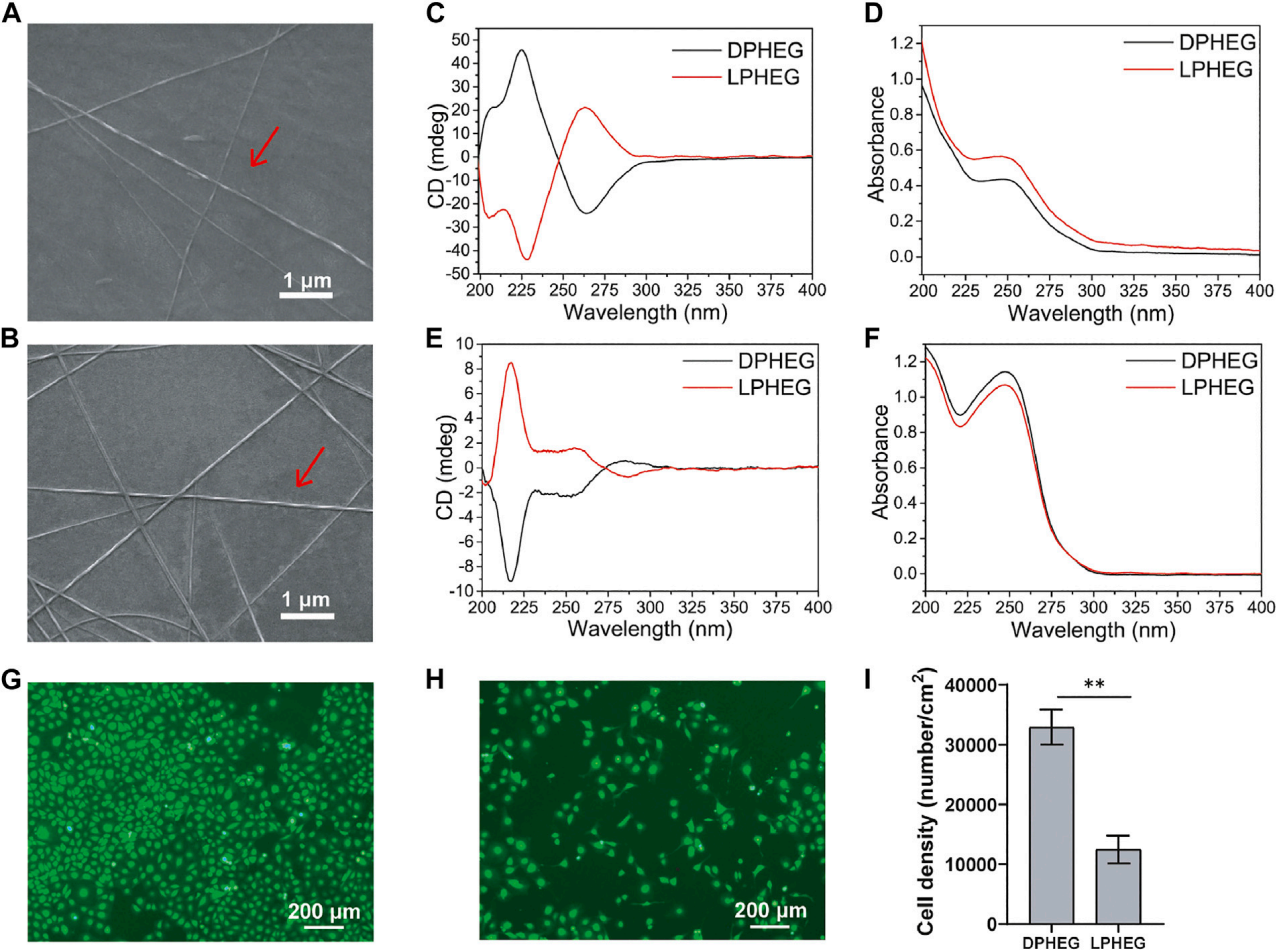
Characterization and cell viability of D(L)PHEG hydrogels. SEM images of **(A)** Right-handed helical DPHEG nanofibers and **(B)** Left-handed helical LPHEG nanofibers. **(C)** CD and **(D)** UV-Vis spectra of DPHEG and LPHEG hydrogels. **(E)** CD and **(F)** UV-Vis spectra of DPHEG HFIP solution and LPHEG HFIP solution. Fluorescence images of HUVECs on **(G)** DPHEG and **(H)** LPHEG hydrogels. Live cells are stained green. **(I)** Cell number of HUVECs on DPHEG and LPHEG hydrogels. ^**^ *p* < 0.01. (*n* = 3).

DPHEG and LPHEG molecules can self-assemble in helical nanofibers in water. The chirality of the hydrogels was characterized by CD and SEM. The SEM images demonstrated that DPHEG and LPHEG self-assembled to form right-handed and left-handed helical structures (Figures 2A,B). The CD/UV-Visible (Vis) absorption spectra of the chiral molecules were quantified (Figures 2C–F). The CD signals of the LPHEG and DPHEG hydrogels in H_2_O showed Cotton effects at 226 and 265 nm (Figures 2C,D), which further confirmed the chirality of DPHEG and LPHEG. In the HFIP solution, DPHEG, and LPHEG molecules also can self-assemble into helical nanofibers. The CD spectra of the DPHEG and LPHEG enantiomers exhibited significant Cotton effects at 217 nm (Figures 2E,F). Furthermore, the shape of the CD spectra changed drastically from one solvent to another, but they all showed chirality.

To explore the influence of the chiral hydrogel on cell spreading and proliferation, HUVECs were cultured on presterilized 24-well plates coated with DPHEG and LPHEG hydrogels. After incubation for 2 days, we found that the number of HUVECs increased (Figures 2G,H; Supplementary Figure S2). The cell densities on the DPHEG and LPHEG hydrogels were (3.29 ± 0.29) × 10^4^/cm^2^ and (1.24 ± 0.23) × 10^4^/cm^2^, respectively, (Figure 2I). The difference in cell density between the two different chiral hydrogels showed that the DPHEG hydrogel could significantly improve cell proliferation, compared with the LPHEG hydrogel.

### Formation of Chiral Hybrid Scaffolds

**FIGURE 3:**
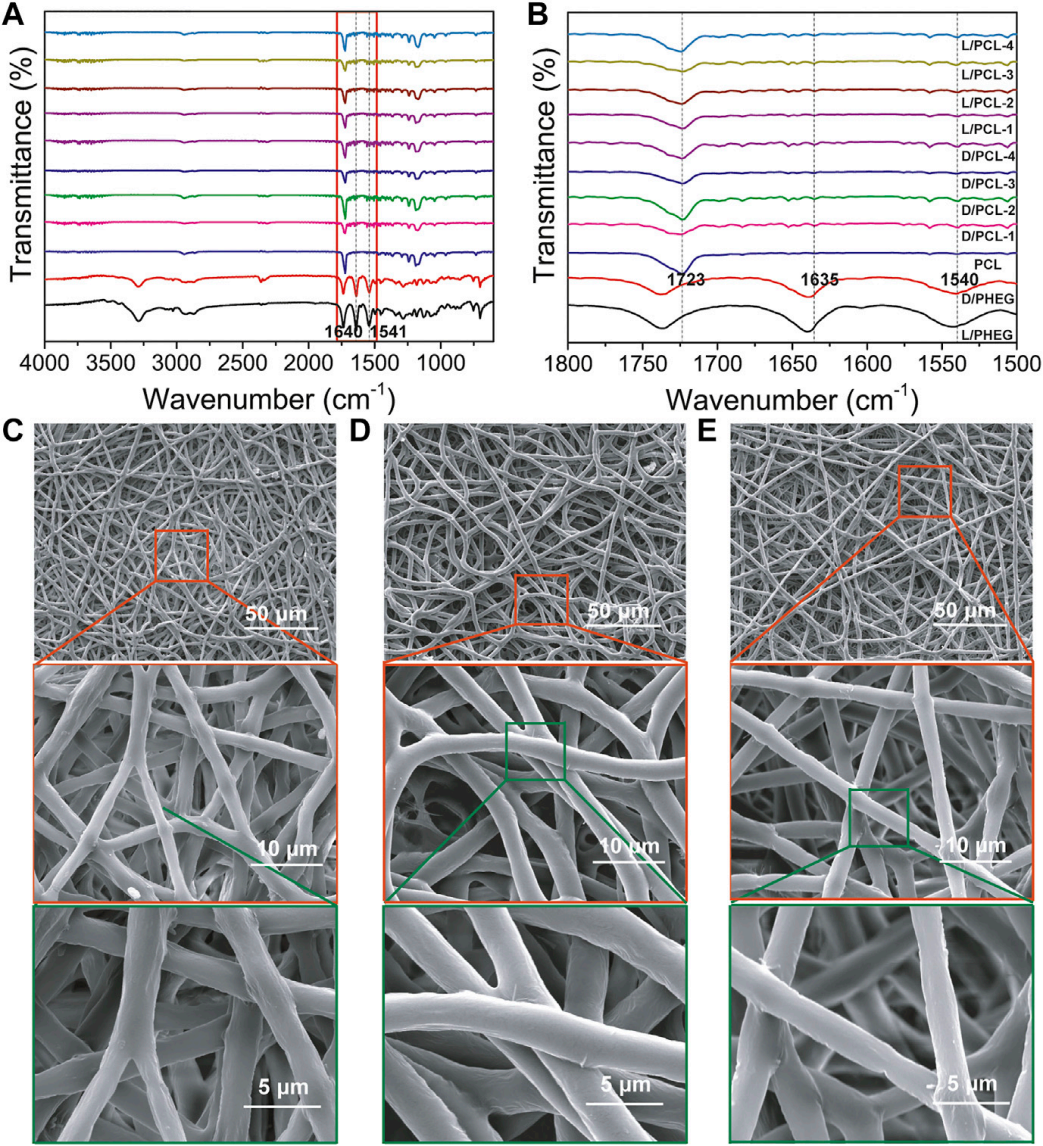
**(A,B)** FTIR spectra of scaffolds. The red rectangle indicates the magnified part. **(B)** the picture of the magnified part. **(C–E)** SEM images of **(C)** PCL, **(D)** D/PCL-2, and **(E)** L/PCL-2 scaffolds.

By blend electrospinning, the resulting chiral hybrid scaffolds at the concentrations of D(L)PHEG (1, 2, 3, and 4 mg/ml) were named as D/PCL-1, D/PCL-2, D/PCL-3, and D/PCL-4 scaffolds, and L/PCL-1, L/PCL-2, L/PCL-3, and L/PCL-4 scaffolds, respectively (Supplementary Figure S3). The PCL scaffold was used as a control. The structure of D(L)PHEG was examined by FTIR spectroscopy (Figure 3A). The D(L)PHEG characteristic absorptions appeared at 1,640 and 1,541 cm^−1^, which can be attributed to the amide I and II bands (Figure 3A). The structures of the resulting scaffolds were characterized by ATR-FTIR. The PCL scaffold was used as the control. The ATR-FTIR characteristic absorption of the PCL scaffold appeared at 1,723 cm^−1^ (Figure 3B) owing to the stretching of the C = O bond in the ester groups that were abundant in the structure of PCL, which also appeared in the spectra of the D/PCL and L/PCL scaffolds. Meanwhile, the ATR-FTIR spectra of the D/PCL and L/PCL scaffolds showed characteristic absorptions at 1,635, 1,540, and 1723 cm^−1^ (Figure 3B), thus revealing the successful integration of D(L)PHEG and PCL. The morphologies of the D/PCL and L/PCL scaffolds were characterized by SEM. We observed similar nanofibrous architectures in all chiral hybrid scaffolds. All scaffolds yielded disordered nanofibers (Figures 3C–E; Supplementary Figure S4).

### Wettability of Chiral Hybrid Scaffolds

**FIGURE 4:**
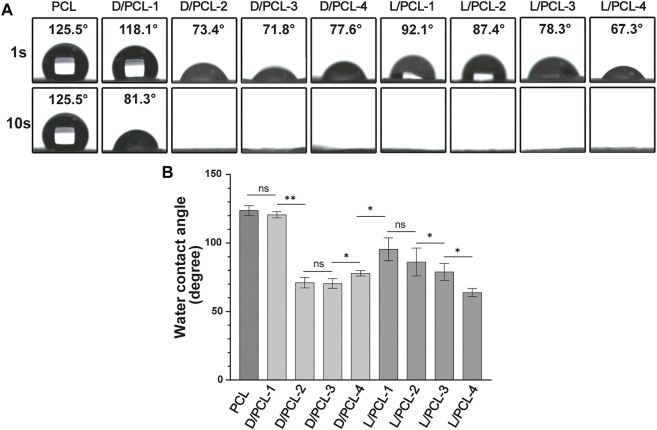
**(A)** Water contact angle of chiral hybrid scaffolds. **(B)** Water contact angle of different scaffolds after 1 s ns means not significant (*p* > 0.05). ^*^ *p* < 0.05. ^**^ *p* < 0.01. (*n* = 3).

Hydrophilicity tests were conducted to determine changes in the wettability of the scaffolds (Figures 4A,B). The PCL scaffold was hydrophobic with a water contact angle of 123.7 ± 3.6 (Figure 4A). In comparison, the hydrophilicities of the D(L)/PCL scaffolds improved because of the addition of LPHEG and DPHEG. After 1 s, the water contact angles of the D/PCL-1, D/PCL-2, D/PCL-3, D/PCL-4, L/PCL-1, L/PCL-2, L/PCL-3, and L/PCL-4 scaffolds decreased slightly to 120.6 ± 2.3, 71.0 ± 3.8, 70.4 ± 3.5, 77.8 ± 2.0, 95.4 ± 8.4, 86.1 ± 10.2, 63.9 ± 3.0, and 78.9 ± 6.2°, respectively (Figure 4A). After 10 s, the contact angles of all the scaffolds (except D/PCL-1 with water contact angle of 85.9 ± 7.6°)decreased to 0°. Finally, the water contact angles of the D/PCL and L/PCL scaffolds were 0° (Supplementary Figure S5). The water contact angles of the D(L)/PCL scaffolds were decreased, which also suggests the successful integration of D(L)PHEG and PCL.

### Mechanical Properties of Chiral Hybrid Scaffolds

**FIGURE 5:**
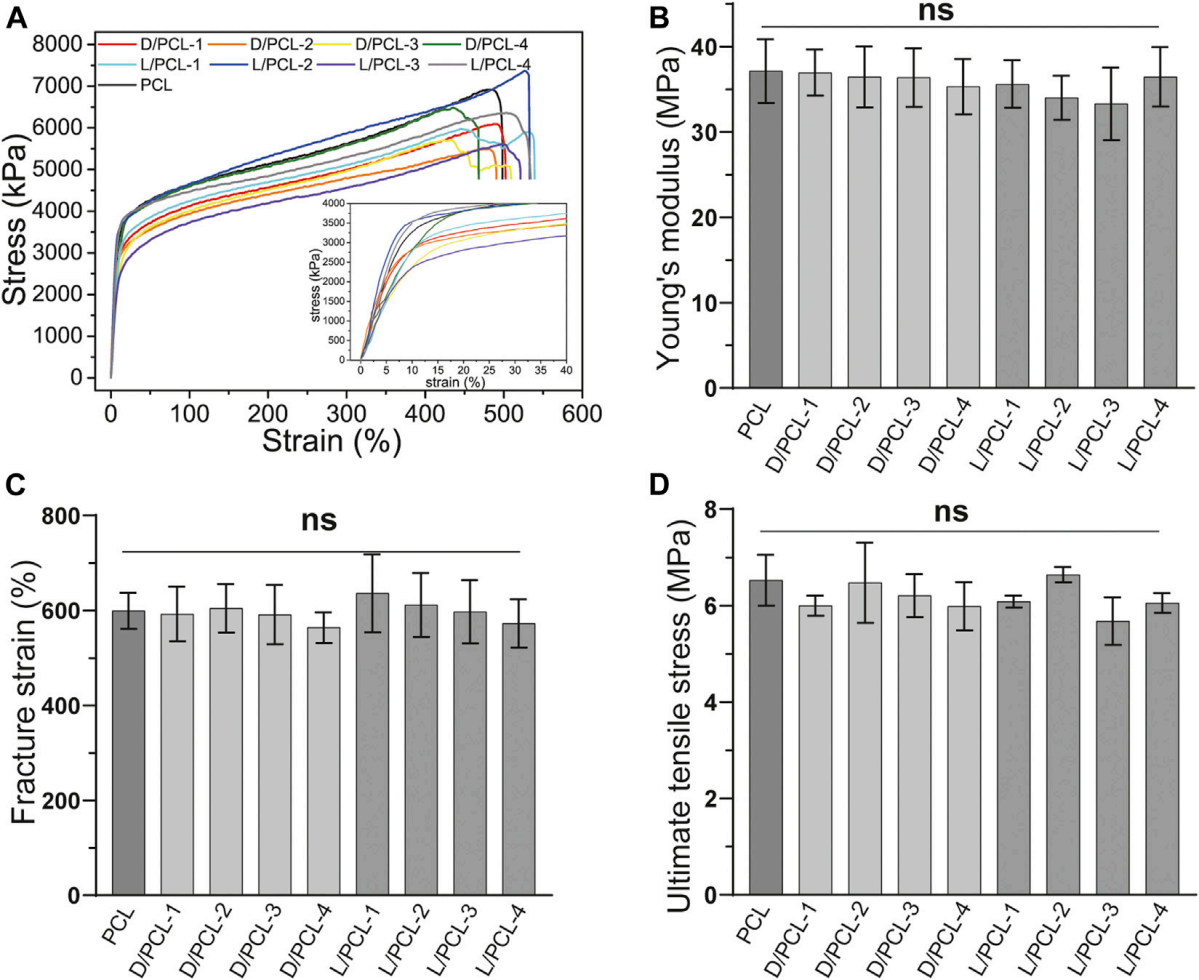
Mechanical properties of chiral hybrid scaffolds. Tensile curves with the inset showing **(A)** the strain-stress curves, **(B)** Young’s modulus, **(C)** fracture strain, and **(D)** ultimate tensile stress of different scaffolds. ns means not significant (*p* > 0.05). (*n* = 3).

 The mechanical properties of tissue-engineered vessels are important for maintaining their structural and functional integrity. The mechanical properties, including Young’s modulus, ultimate tensile stress, and fracture strain of different chiral hybrid scaffolds were evaluated by tensile tests. All scaffolds displayed similar mechanical characteristics (Figure 5). PCL scaffolds yielded a Young’s modulus of 37.17 ± 3.74 MPa (Figure 5B), fracture stain of 599.45 ± 38.32% (Figure 5C) and ultimate tensile stress of 6.53 ± 0.53 MPa (Figure 5D). Compared with the PCL scaffold, the chiral hybrid scaffolds L/PCL-1, L/PCL-2, L/PCL-3, L/PCL-4, D/PCL-1, D/PCL-2, D/PCL-3, and D/PCL-4 scaffolds exhibited similar Young’s modulus, fracture strain, and ultimate tensile stress outcomes (Figures 5B–D).

### The Effect of Chiral Hybrid Scaffolds on Endothelium Remodeling

**FIGURE 6:**
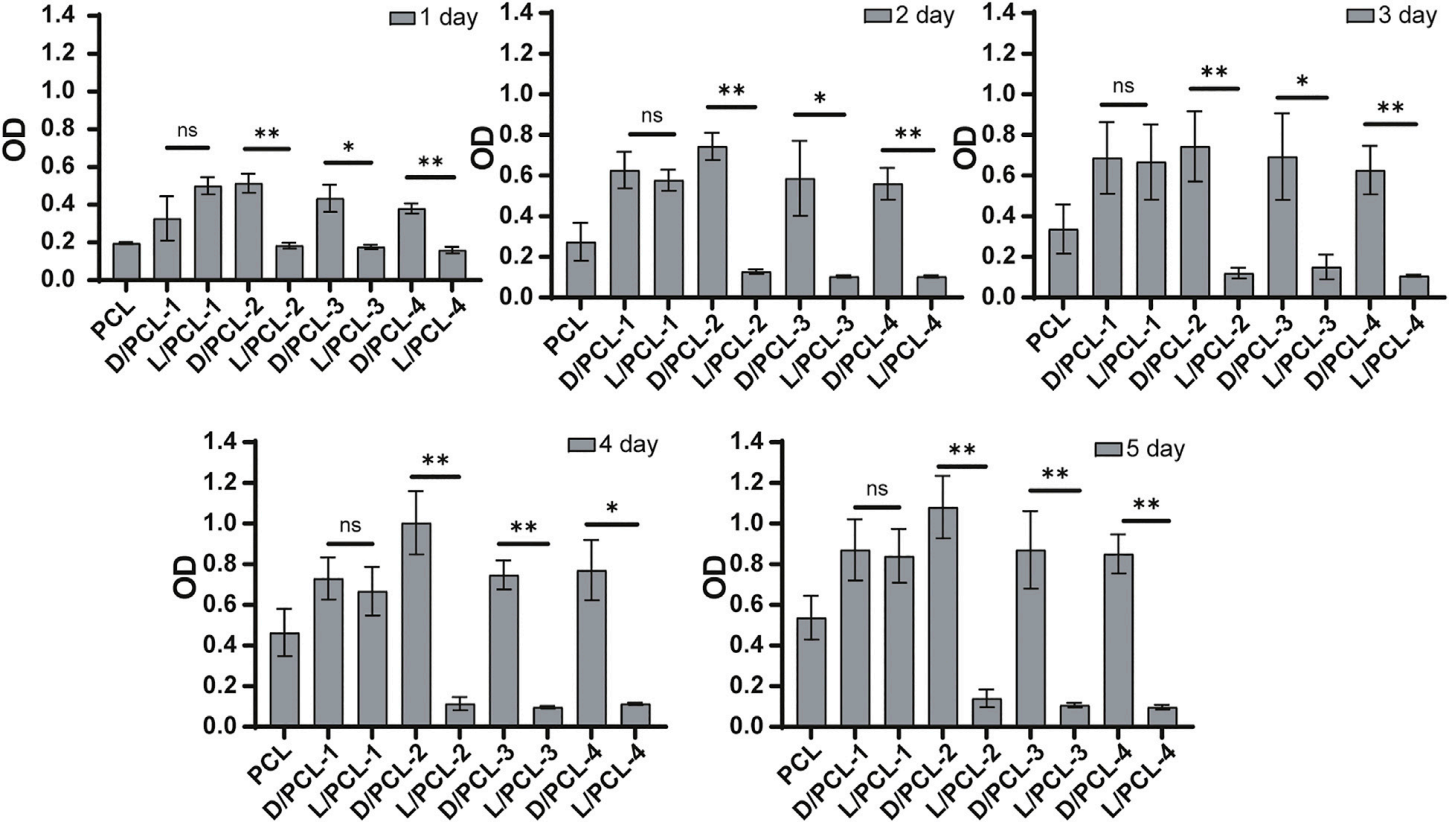
CCK-8 assay results of HUVECs cultured on chiral hybrid scaffolds after 1–5 days ns means not significant (*p* > 0.05). ^*^ *p* < 0.05. ^**^ *p* < 0.01. (*n* = 3).

We found that the cell density of the DPHEG hydrogel was higher than that of the LPHEG hydrogel. Chirality is an important influencing factor for the endothelial remodeling of TEVGs (Figure 2I). To further explore the influence of chiral hybrid scaffolds with chiral molecules on endothelium remodeling, HUVECs were incubated on PCL, D/PCL-1, D/PCL-2, D/PCL-3, D/PCL-4, L/PCL-1, L/PCL-2, L/PCL-3, and L/PCL-4 scaffolds. The adhesion and proliferation of HUVECs were tested using the CCK-8 assay and fluorescence staining. As depicted in Figure 6, HUVECs could adhere and proliferate on PCL, D/PCL-1, D/PCL-2, D/PCL-3, D/PCL-4, and L/PCL-1 scaffolds. The number of HUVECs increased slightly after successive 24-h periods (Figure 6). Therefore, on D/PCL scaffolds, the number of cells was relatively higher than those on L/PCL and PCL scaffolds. The number of cells on D/PCL-2 was greater than those on the other scaffolds. When the concentration of DPHEG was >2 mg/ml, compared to that of D/PCL scaffolds, the number of cells decreased as the DPHEG level increased. However, for L/PCL scaffolds, cells adhered to and proliferated only on L/PCL-1 scaffolds.

**FIGURE 7:**
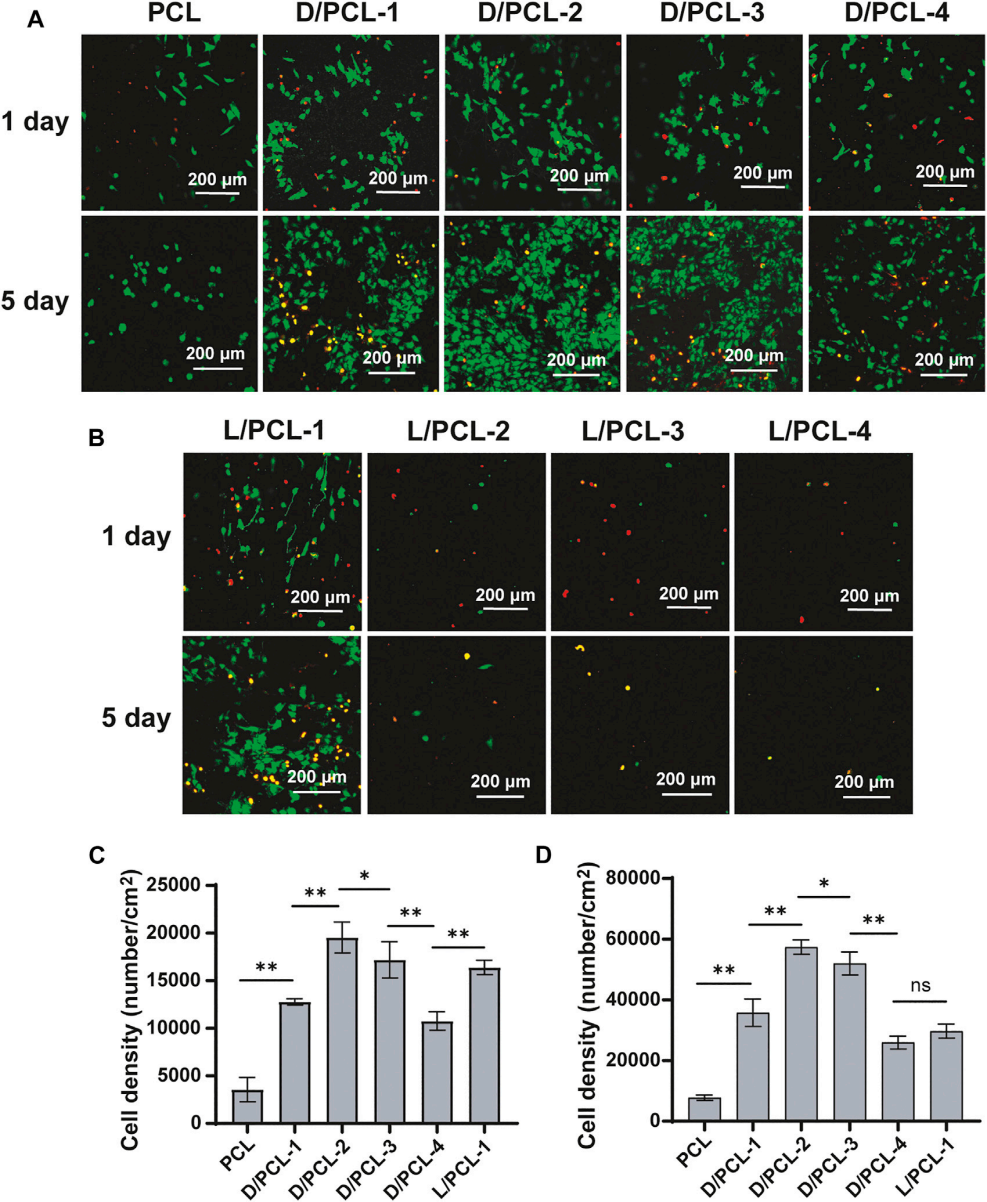
**(A** *,* **B)** Confocal laser scanning microscope images of HUVECs after 1 and 5 days cultured on chiral hybrid scaffolds. Live cells and dead cells were stained green and red, respectively. **(C)** Average spreading area per cell for HUVECs cultured on chiral hybrid scaffolds after 1 day **(D)** Average spreading area per cell for HUVECs cultured on different scaffolds after 5 days ns means not significant (*p* > 0.05). ^*^ *p* < 0.05. ^**^ *p* < 0.01. (*n* = 3).

Subsequently, a similar finding was observed; the cell density on D/PCL scaffolds was higher than that on L/PCL scaffolds based on fluorescence staining (Figures 7A,B). After 1 day of culture, the average number of cells on the PCL, D/PCL-1, D/PCL-2, D/PCL-3, D/PCL-4, and L/PCL-1 scaffolds were (3.56 ± 1.28) × 10^3^/cm^2^, (1.27 ± 0.03) × 10^4^/cm^2^, (1.95 ± 0.16) × 10^4^/cm^2^, (1.71 ± 0.19) × 10^4^/cm^2^, (1.07 ± 0.10) × 10^4^/cm^2^, and (1.64 ± 0.08) × 10^4^/cm^2^, respectively, (Figure 7C). The D/PCL-2 scaffold possessed the highest cell density. The cell density of the PCL scaffold was the lowest. Compared with the findings on day 1, the number of cells increased slightly after day 5. The cell densities of the PCL, D/PCL-1, D/PCL-2, D/PCL-3, D/PCL-4, and L/PCL-1 scaffolds were (7.75 ± 0.90) × 10^3^/cm^2^, (3.58 ± 0.45) × 10^4^/cm^2^, (5.74 ± 0.24) × 10^4^/cm^2^, (5.20 ± 0.38) × 10^4^/cm^2^, (2.59 ± 0.21) × 10^4^/cm^2^ and (2.97 ± 0.23) × 10^4^/cm^2^, respectively, (Figure 7D). Similarly, the D/PCL-2 scaffold possessed the highest cell density. The fluorescence staining images demonstrated a higher HUVEC density when the cells were grown on D/PCL scaffolds compared with those on the L/PCL or PCL scaffolds. Among them, the D/PCL-2 scaffold yielded the highest cell density.

**FIGURE 8:**
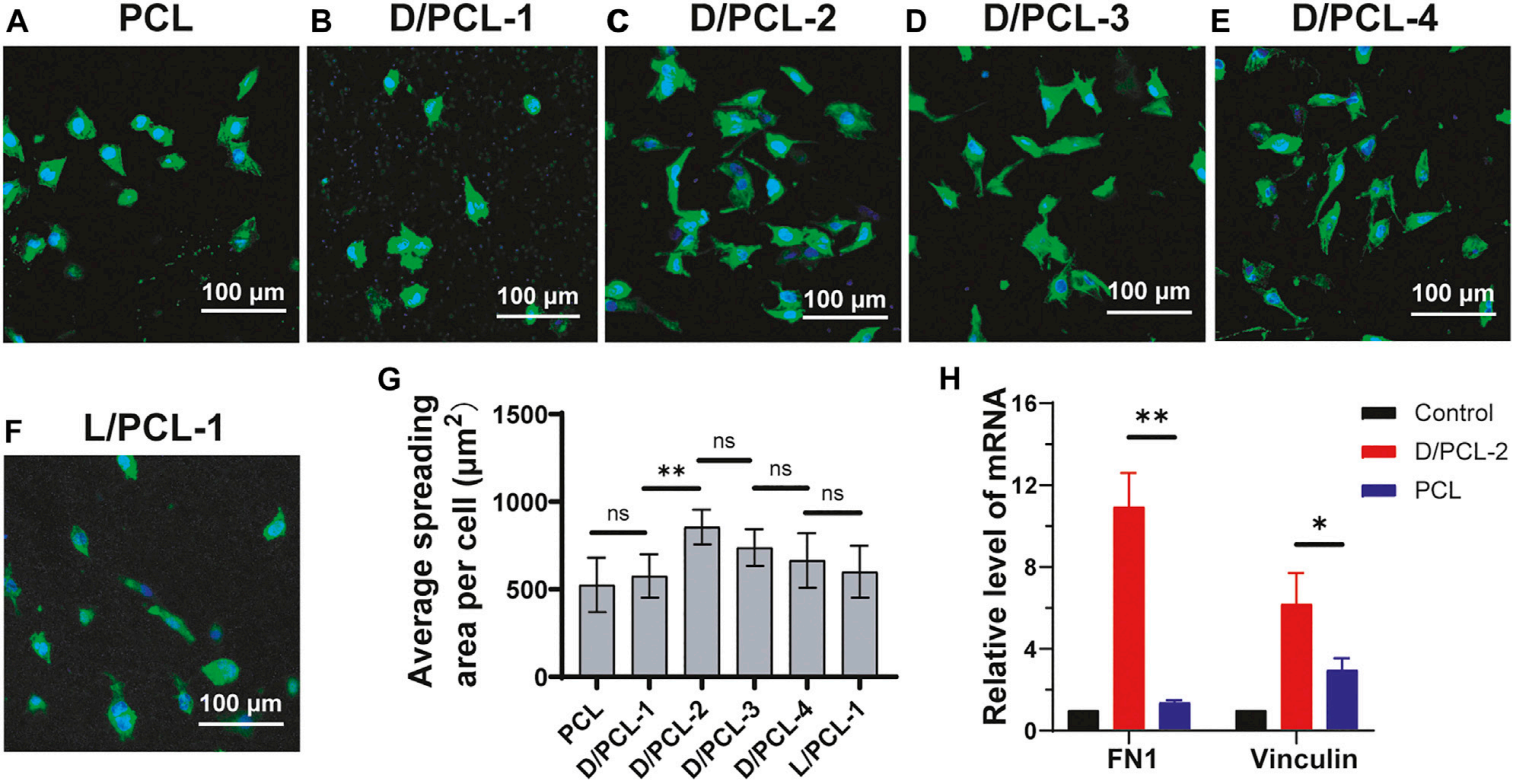
**(A–F)** Confocal laser scanning microscope images of HUVECs after 5-days cultured on **(A)** PCL, **(B)** D/PCL-1, **(C)** D/PCL-2, **(D)** D/PCL-3, **(E)** D/PCL-4, and **(F)** L/PCL-1 scaffolds. Actin and nuclei were stained into green and blue, respectively. **(G)** for HUVECs cultured on different scaffolds. (*n* = 5). **(H)** RT-qPCR analysis of expression levels of FN1and vinculin in differentially treated 7-days cultures. ns means not significant (*p* > 0.05). ^**^ *p* < 0.01. (*n* = 3).

After 1 day of culture, the cells on PCL, D/PCL-1, D/PCL-2, D/PCL-3, D/PCL-4, and L/PCL-1 scaffolds appeared polygonal in shape and attached well, as documented by confocal laser microscopy (Figures 8A–F). The average cell spreading areas per cell were 524.76 ± 155.48, 526.84 ± 129.24, 855.67 ± 99.23, 737.94 ± 104.92, 665.43 ± 155.62, and 599.89 ± 148.23 μm^2^ (Figure 8G). The cells on the D/PCL-2 scaffolds had the largest average spreading area.

The aforementioned results indicate that chirality was an important influencing factor for endothelial remodeling, and HUVECs had the highest cell viability in the D/PCL-2 scaffold. Therefore, the D/PCL-2 scaffold was selected for the TEVGs. To detect how the D/PCL-2 scaffold influences the process of endothelial remodeling, HUVECs were seeded on a D/PCL-2 scaffold to detect relative protein expression levels using RT-qPCR. Fibronectin-1 (FN-1) and vinculin are two well-known proteins that promote cell adhesion (Bays and DeMali, 2017; Sun et al., 2020; Yang et al., 2020). FN-1 and vinculin were upregulated in the D/PCL-2 scaffold (Figure 8H), which suggests that the D/PCL-2 scaffold promoted cell adhesion and proliferation by enhancing the two proteins. In addition, we measured Bcl-2 and caspase-3 and found that the two proteins did not increase (Supplementary Figure S6).

## Discussion

Tissue engineering for transplantation is a rapidly developing regenerative therapy field (Piard et al., 2019). Improved methods for fabricating TEVGs have been reported (Andrique et al., 2019). Foreign surfaces may lead to stenosis and thrombosis. Therefore, an integrated and well-functional endothelium is a critical factor for tissue-engineered vascular grafts (Seifu et al., 2013). The endothelial remodeling of TEVGs was regulated by many factors. The materials used for TEVGs play an important role in this process. The materials must have ideal physicochemical and mechanical properties as vascular grafts directly contact the blood. In this study, chiral hybrid scaffolds were constructed by blending electrospinning using PCL and the chiral molecule D(L)PHEG. The PCL component of the scaffolds can provide mechanical properties to maintain structural integrity, but its ion-inert properties limit its extensive use in vascular regeneration (Rafique et al., 2021). To overcome the inert property, PCL is always used with other biomaterials for functional modification (Yue et al., 2015; Yang et al., 2019). D(L)PHEG molecules can modify the biological activity of scaffolds. Chirality is also a vital factor that affects the endothelial remodeling process. First, we investigated the effects of chiral hydrogels on cell behavior. Cell culture results demonstrated that by changing the chirality of the hydrogel, HUVECs expressed different growth abilities. From the results (Figures 2G,H), we found that two types of chiral hydrogels exhibited appropriate good compatibility, and the number of cells on the DPHEG hydrogel was almost two times higher than that on the LPHEG hydrogel. This showed that chirality can influence cell adhesion and proliferation.

Subsequently, we explored the effect of chirality on the endothelial remodeling process induced by TEVGs. The effect of chiral hybrid scaffolds on the regulatory mechanism of cell behaviors and endothelium remodeling are complicated (Khan and Sefton, 2011). All the scaffolds were fabricated by electrospinning. Electrospun nanofiber scaffolds can enhance cell adhesion, spreading, and proliferation owing to their morphologies (Kang et al., 2019). The resulting chiral hybrid scaffolds possessed nanofibers with similar degrees of disorder. The morphology of nanofibers is an important factor in manipulating the elongation and aligned growth of cells (Wang et al., 2020). In addition, all scaffolds had the same mechanical properties, implying similar stiffnesses. Stiffness is also a critical factor that influences cell growth (Yi et al., 2019). Therefore, similar nanofiber structures of different scaffolds yielded the same effects on cells. The different concentrations of D(L)PHEG had little influence on the structure and mechanical properties of the chiral hybrid scaffolds.

While the topographies of different scaffolds exhibited minor differences, the wettability and bioactivity properties were tuned based on the scaffold components. The successful integration of PCL and D(L)PHEG hydrogels increased the hydrophilicity of the electrospun scaffolds owing to the water solubility of the chiral hydrogel. The adhesion and proliferation of cells on scaffolds may be enhanced by increased hydrophilicity (Sharon L. Sanborna et al., 2002). The PCL scaffold was fabricated as the control. The aforementioned results confirm that HUVECs can grow on PCL, D/PCL-1, D/PCL-2, D/PCL-3, D/PCL-4, and L/PCL-1 scaffolds. Compared with PCL scaffolds, chiral hybrid scaffolds are more conducive to cell growth. In the scaffolds on which HUVECs could adhere and proliferate, the PCL scaffold exhibited the lowest cell density and minimum spread area. On different scaffolds, HUVEC behavior was tuned by varying the compositions of scaffolds using different concentrations of D(L)PHEG molecules. Compared with L/PCL scaffolds, cells expressed better growth patterns on D/PCL scaffolds. FN-1 and vinculin expressions in HUVECs on the D/PCL-2 scaffold cultured for 7 days was higher than that on the PCL scaffold. By enhancing the two focal adhesion-related proteins, HUVECs seeded on D/PCL scaffolds displayed higher cell growth status. The detection of apoptosis markers confirmed that the cells did not undergo apoptosis from days 1 to 7. As the concentration of LPHEG molecules increased, the cells hardly survived. According to the CCK-8 assay, it is clear that the number of cells did not increase proportionally with respect to the linear increase in D(L)PHEG hydrogel concentration in chiral hybrid scaffolds. We hypothesized that as the concentration of the chiral molecule continued to increase, the molecule exhibited a specific level of cytotoxicity. For LPHEG concentration was >1 mg/ml, L/PCL scaffolds were highly cytotoxic. Because D/PCL scaffolds can upregulate adhesion-related proteins, they require higher concentrations to inhibit cell adhesion and proliferation. D/PCL scaffolds show cytotoxicity until the concentration of DPHEG is > 3 mg/ml. Figures 8A–G demonstrates that, cells exhibited excellent stretched cell morphology and cell adhesion patterns compared with other scaffolds on the D/PCL-2 scaffold. D/PCL-2 possessed the largest spreading area, which was 1.6 times larger than that on the PCL scaffold. The aforementioned results demonstrated that chiral molecules contained in hybrid scaffolds had a substantial impact on endothelial remodeling. Among these scaffolds, the D/PCL-2 scaffold possessed the highest number of cells and the largest spreading area. Therefore, the D/PCL-2 scaffold was selected as the ideal scaffold for the fabrication of TEVGs.

As discussed above, both the chiral hydrogel and hybrid scaffolds contained chiral molecules, which can influence the cell status. Chirality is a common effect that influences different cell lines that regulate cell behavior during endothelial remodeling. By adding chiral molecules, fabricating hybrid scaffolds can serve as a promising method for tissue-engineered vessels. In this study, we focused on the exploration of the influence of chiral hybrid scaffolds on cell behavior. However, the specific regulatory mechanism of chiral hybrid scaffolds that regulate endothelial remodeling remains unclear. The effect of chirality on other cell lines still needs to be explored.

## Conclusion

In conclusion, we fabricated D/PCL and L/PCL scaffolds with D(L)PHEG and PCL by blending electrospinning to detect the moderating effect of chiral structures that play a crucial role in vascular endothelium remodeling. Compared with L/PCL scaffolds, D/PCL scaffolds showed better regulation of cell adhesion, spreading, and proliferation. The D/PCL-2 scaffold significantly promoted endothelial remodeling by enhancing FN-1 and vinculin. The chiral hybrid scaffolds are promising materials used to aid desirable endothelium remodeling, which is a critical step for the success of TEVGs. This study was an attempt to utilize chiral hybrid scaffolds to fabricate tissue-engineered vessels for the treatment of cardiovascular diseases. The study also revealed that chirality is a vital factor that ought to be considered in tissue engineering.

## Data Availability

The original contributions presented in the study are included in the article/Supplementary Material, further inquiries can be directed to the corresponding authors.
