# Supplementary material for: Use of Electrospun Phenylalanine/Poly-ε-Caprolactone Chiral Hybrid Scaffolds to Promote Endothelial Remodeling
Source: Front Bioeng Biotechnol. 2021 Nov 25;9:773635. doi: 10.3389/fbioe.2021.773635 (PMC8656108; doi:10.3389/fbioe.2021.773635)
Supplement: Supplementary file 1 [file DataSheet1.docx]

**Supplementary Material**


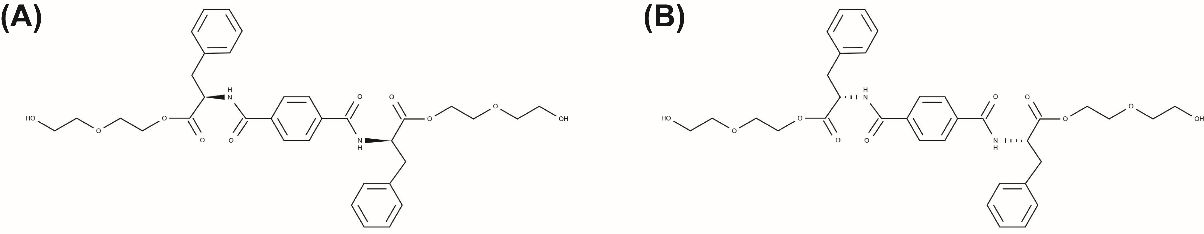


**Figure S1.** Molecular structures of **(A)** DPHEG and **(B)** LPHEG.


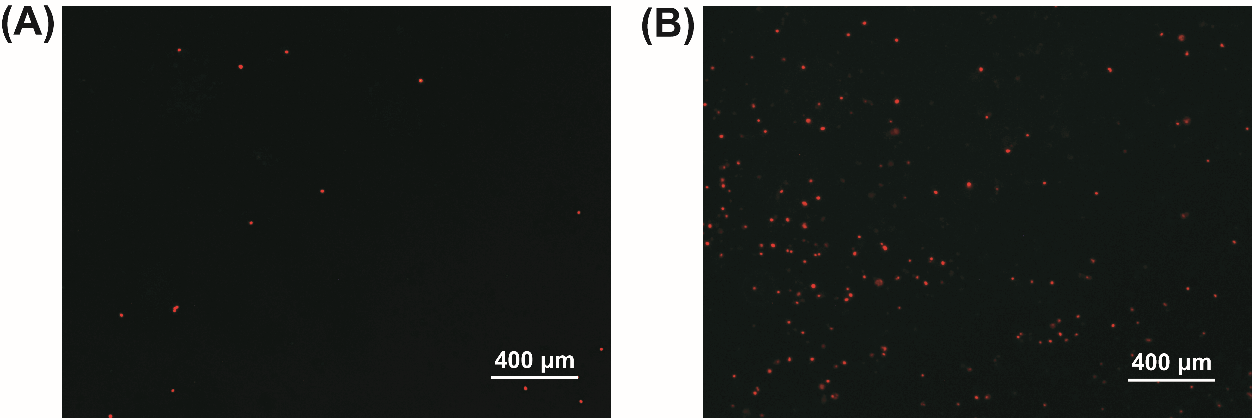


**Figure S2.** Fluorescence images of HUVECs on **(A)** DPHEG and **(B)** LPHEG hydrogel. Dead cells were stained red.


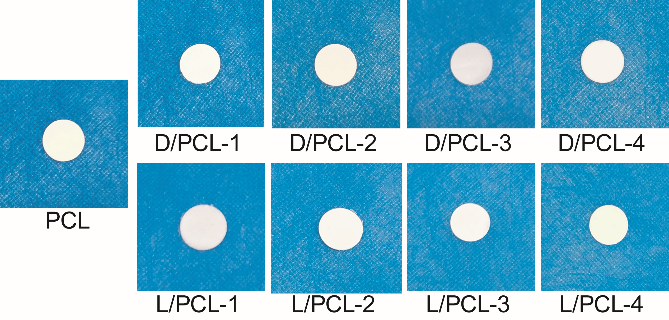


**Figure S3**. Photographic images of scaffolds cut into suitable shape.


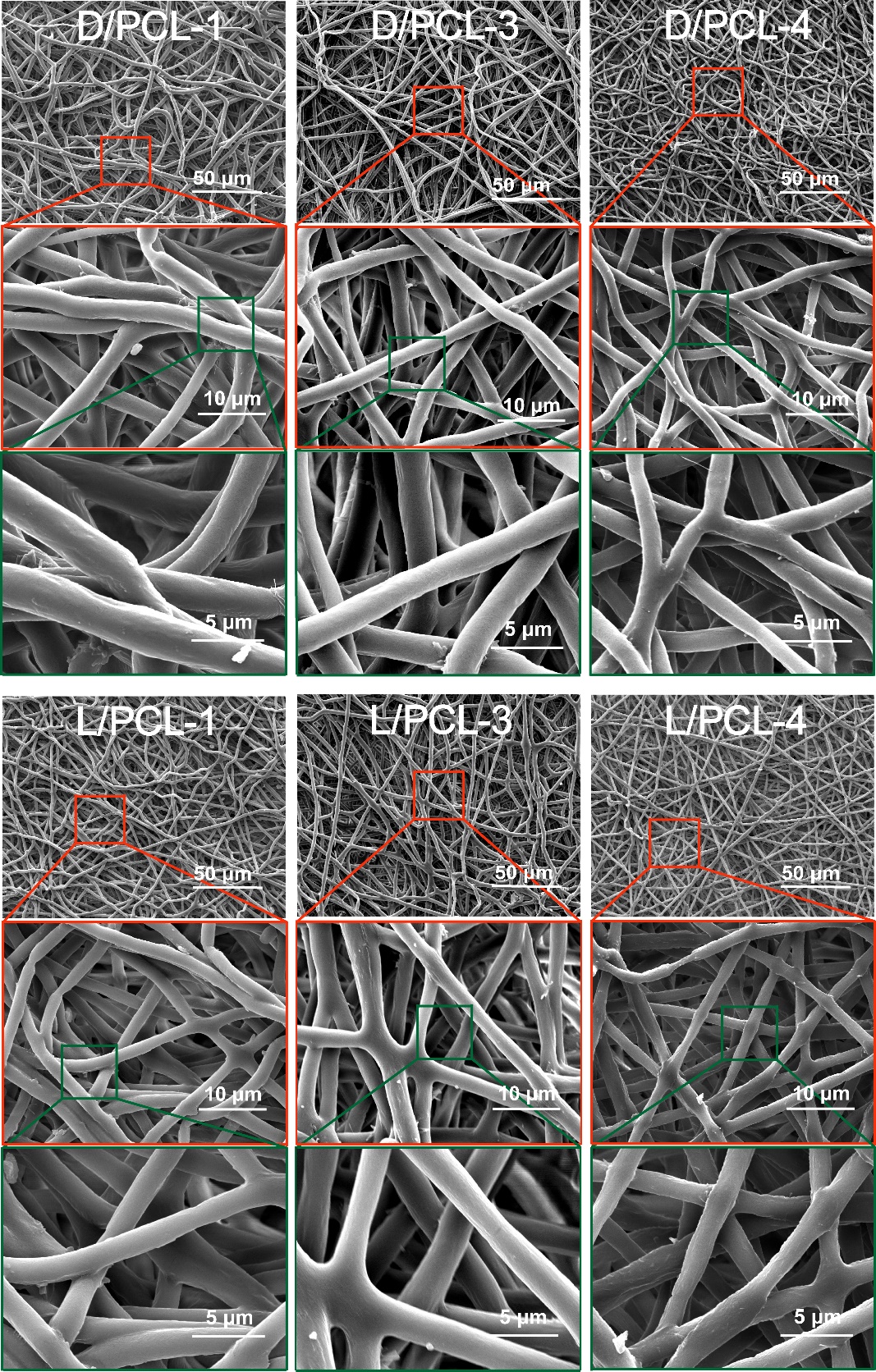


**Figure S4.** SEM images of D/PCL-1, D/PCL-3, D/PCL-4, L/PCL-1, L/PCL-3, and L/PCL-4 scaffolds.


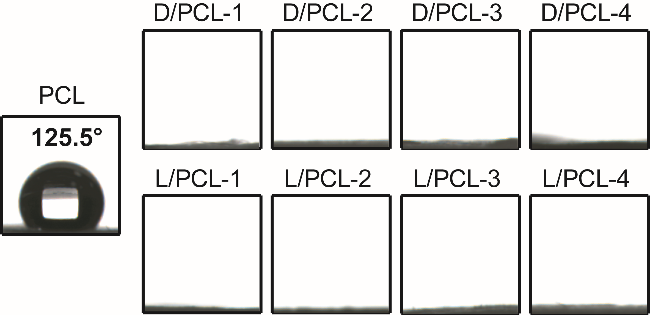


**Figure S5.** Static water contact angles of different chiral hybrid scaffolds.


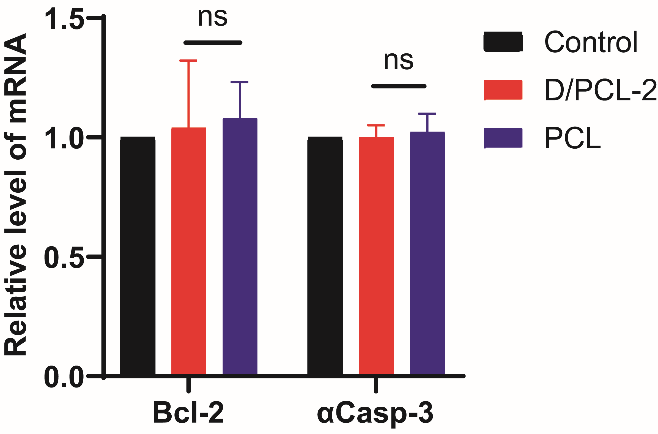


**Figure S6.** RT-qPCR analysis of expression levels of Bcl-2 and αCasp-3 in differentially treated 7-day cultures. ns means not significant (p > 0.05). Error bars: standard error (n = 3).

**Table S1.** Primers used for quantitative real-time PCR analysis.

| Target gene | Forward sequence (5’-3’) | Reward sequence (3’-5’) |
| --- | --- | --- |
| Bcl-2 | TGTGTGTGGAGAGCGTCAAC | CTCAGCCCAGACTCACATCA |
| FN1 | TCAGCTTCCTGGCACTTCTG | TCTTGTCCTACATTCGGCGG |
| Vinculin | ATTCCACTTCTCTGTCGCCC | GCCCTCCTCGTGCATTATCA |
| αCasp-3 | ATGTCCTGGGACACCGGTTA | TGAGGTTTGCTGCATCGACA |
| GAPDH | GGTGAAGGTCGGAGTCAACG | CTTCCCGTTCTCAGCCATGTA |
